# A compartment-resolved framework for microbial sulfur–reactive sulfur signaling in diabetic cardiomyopathy: from gut metabolism to cardiac sulfur responses

**DOI:** 10.3389/fmicb.2026.1966245

**Published:** 2026-09-29

**Authors:** Mingda Han, Xiao Sun, Yue Mu, Jiarui Li, Yue Zhang, Hao Xu, Jinyue Zhao, Shuang Zhao, Kaile Ma, Jing Jia, Min Li

**Affiliations:** 1 College of Traditional Chinese Medicine, Changchun University of Chinese Medicine, Changchun, China; 2 China Academy of Chinese Medical Sciences Guang'anmen Hospital, Beijing, China; 3 Affiliated Hospital to Changchun University of Chinese Medicine, Changchun, China

**Keywords:** diabetic cardiomyopathy, gut microbiota, gut-heart axis, microbial sulfur metabolism, protein persulfidation, reactive sulfur signaling

## Abstract

Diabetic cardiomyopathy (DCM) is an important substrate of diabetes-related heart failure and is increasingly considered within a gut–heart systems framework. Here, we propose a compartment-resolved microbial sulfur–cardiac signaling model rather than a linear pathway in which free gut-derived sulfide species are assumed to reach the myocardium intact. Gut microbes generate sulfide through dissimilatory sulfate reduction, anaerobic sulfite reduction, assimilatory sulfur pathways, and sulfur-amino-acid catabolism, whereas intestinal epithelial cells and liver strongly constrain systemic exposure through mitochondrial sulfide oxidation. Recent work reports that microbial sulfide-associated metabolism can influence enteroendocrine GLP-1 signaling, hepatic PPARα-dependent metabolism, and systemic sulfur handling through intestinal SQOR-dependent pathways; however, none of these studies demonstrates direct sulfur transfer from microbial metabolism to the diabetic heart or establishes a sulfur-specific causal pathway leading to myocardial persulfidation. In parallel, diabetic-heart studies report sulfide-donor-responsive persulfidation candidates including MuRF1 Cys44, SYVN1/Hrd1 Cys115, and SENP1 Cys683, but the evidence is concentrated in an overlapping research program and is methodologically dependent. We therefore separate better-supported local processes, cross-organ supporting evidence, and unresolved DCM-specific links, and outline source-resolved isotope tracing, microbial genetics, host sulfur-enzyme models, and orthogonal site-resolved persulfidation assays needed to test causality.

## Introduction

Diabetic cardiomyopathy (DCM) refers to myocardial structural and functional abnormalities in diabetes that cannot be fully explained by coronary artery disease, hypertension, or valvular disease. Its pathobiology includes metabolic inflexibility, mitochondrial dysfunction, oxidative stress, inflammation, cell death, and interstitial fibrosis, culminating in diastolic and/or systolic dysfunction (Jia et al., 2018; Tan et al., 2020; Borghetti et al., 2018). Type 2 diabetes is also accompanied by reproducible compositional and functional changes in the gut microbiome, and microbiota-transfer studies indicate that intestinal ecosystems can modify host metabolic phenotypes (Qin et al., 2012; Vrieze et al., 2012). Recent multi-omics and intervention studies further implicate microbiota-associated metabolites in diabetic cardiac remodeling (Shi et al., 2025; Zheng et al., 2024; Zhu et al., 2024).

Hydrogen sulfide (H₂S) is a plausible connector between microbial metabolism and cardiac sulfur signaling, but the relevant chemistry is source- and species-dependent. Host tissues generate H₂S through cystathionine-γ-lyase (CSE), cystathionine-β-synthase (CBS), and 3-mercaptopyruvate sulfurtransferase (3-MST), whereas gut microbes use multiple inorganic- and organic-sulfur pathways (Shen et al., 2013; Braccia et al., 2021; Wolf et al., 2022; Luo et al., 2024). Protein persulfidation converts a cysteine sulfur center to a persulfide (-SSH), but free H₂S/HS^−^ cannot simply add sulfur to a reduced cysteine thiolate. Plausible routes include reactions with oxidized cysteine intermediates, reactive sulfane-sulfur species such as polysulfides, radical chemistry, and enzymatic transpersulfidation; reaction rates and low steady-state sulfide constrain the contribution of disulfide reactions *in vivo* (Zhang et al., 2014; Filipovic et al., 2018). Accordingly, “H₂S signaling” is used here as shorthand for a broader reactive-sulfur network rather than as proof that free H₂S is the direct modifying species.

The conceptual contribution of this Mini Review is a compartment-resolved sulfur relay framework that integrates microbial sulfur metabolism, intestinal and hepatic sulfur processing, candidate systemic mediators, endogenous cardiac sulfur metabolism, and myocardial reactive-sulfur signaling. Unlike a simple gut-derived H₂S delivery model, this framework emphasizes that microbial sulfur metabolism may influence cardiac sulfur signaling through multiple chemically distinct routes, which remain to be experimentally connected in diabetic cardiomyopathy. These processes are individually supported to different degrees, but the available studies do not establish a continuous sulfur-specific causal pathway in DCM. The key testable alternative to a simple “gut H₂S reaches the heart” model is that microbial sulfur metabolism may alter cardiac reactive-sulfur signaling without appreciable delivery of intact free H₂S (Figure 1).

**Figure 1:**
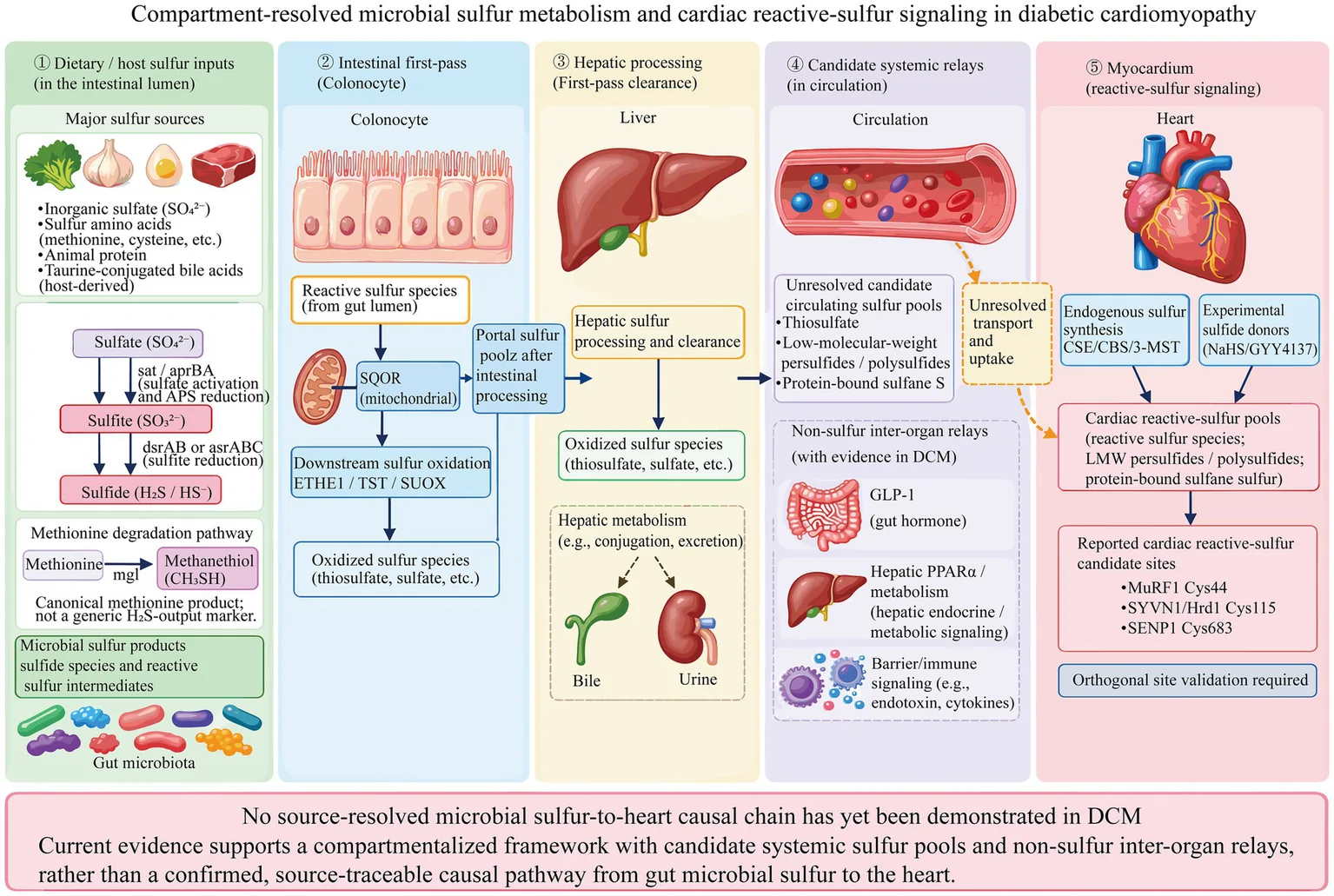
Compartment-resolved framework for microbial sulfur metabolism and cardiac reactive-sulfur signaling in diabetic cardiomyopathy. The five numbered panels distinguish (1) microbial sulfur production from dietary and host-derived substrates in the intestinal lumen; (2) intestinal first-pass sulfide oxidation in colonocytes, involving SQOR, ETHE1, TST/rhodanese, and SUOX; (3) hepatic sulfur processing and clearance; (4) candidate circulating sulfur pools and non-sulfur inter-organ relays; and (5) myocardial reactive-sulfur signaling, including endogenous sulfur synthesis, experimental sulfide-donor exposure, and reported candidate sites. Solid arrows indicate experimentally supported local processes or interventions, whereas dashed arrows indicate proposed or unresolved connections. The figure does not imply that microbial free H₂S directly reaches the myocardium or directly modifies cardiac cysteine residues. Instead, microbial sulfur metabolism may influence cardiac reactive-sulfur signaling through chemically distinct sulfur pools, host metabolic adaptation, endocrine pathways, inflammatory signals, or regulation of endogenous cardiac sulfur synthesis. MuRF1 Cys44, SYVN1/Hrd1 Cys115, and SENP1 Cys683 represent reported cardiac reactive-sulfur candidate sites with methodological limitations and incomplete independent validation. CBS, cystathionine-β-synthase; CSE, cystathionine-γ-lyase; DCM, diabetic cardiomyopathy; ETHE1, persulfide dioxygenase; GLP-1, glucagon-like peptide-1; 3-MST, 3-mercaptopyruvate sulfurtransferase; SQOR, sulfide:quinone oxidoreductase; SUOX, sulfite oxidase; TST, thiosulfate sulfurtransferase.

## Gut microbial sulfur metabolism: genes, substrates, and flux

Intestinal sulfidogenesis is not a single pathway. Classical dissimilatory sulfate reduction (DSR) uses sulfate as a terminal electron acceptor: *sat* encodes sulfate activation, *aprBA* encodes adenosine-5′-phosphosulfate reduction, and *dsrAB* encodes dissimilatory sulfite reduction. *Desulfovibrio* is a canonical gut DSR guild (Rey et al., 2013; Wolf et al., 2022). Anaerobic sulfite reduction through *asrABC* provides a distinct route across gut taxa. Assimilatory sulfate reduction can also contribute to H₂S output in specific contexts; deletion of *cysJ* or *cysM* altered bacterial sulfidogenic capacity in a Crohn’s disease study (Luo et al., 2024). Gene presence alone establishes neither transcriptional activity nor net sulfur flux.

Organic sulfur metabolism further broadens the source space. Cysteine-degrading enzymes encoded by *dcyD* and *malY*, together with *metC*-related lyase activities, can release H₂S. By contrast, *cysK*/*cysM* primarily encode cysteine synthesis, whereas *iscS*/*sufS* participate in sulfur transfer; substrate-dependent side activities complicate their use as output markers (Braccia et al., 2021; Wolf et al., 2022). Methionine γ-lyase (*mgl*) canonically produces methanethiol from methionine, so it is not a generic direct H₂S marker. Braccia et al. (2021) detected expression of putative cysteine-degrading genes in human gut metatranscriptomes, but these observations do not establish their differential expression or net sulfur output in type 2 diabetes (T2D). Current T2D microbiome studies mainly provide taxonomic or community-level functional associations; direct measurements linking sulfur gene expression, substrate availability, and sulfur flux remain lacking. Enzyme–substrate–product resolution is therefore essential.

Diet and host secretions determine which pathways are used. Dietary sulfate and sulfur-containing amino acids supply substrates, whereas taurine-conjugated bile acids can select for organisms specialized in organosulfur respiration. *Bilophila wadsworthia* is therefore better described as a taurine-associated sulfite respirer than as a canonical sulfate reducer; milk-fat-driven increases in taurine-conjugated bile acids can promote its expansion (Devkota et al., 2012). These diet–bile-acid–microbial interactions make sulfur input and substrate availability integral variables in any DCM study of microbial sulfide production.

Diabetes-specific sulfur evidence remains suggestive rather than flux-resolved. Qin et al. (2012) reported enrichment of sulfate-reduction-related functions in T2D metagenomes. Mei et al. (2024) identified strain-level and community-functional associations in 8,117 metagenomes, supporting the broader T2D microbiome context rather than a demonstrated sulfur flux. More recently, Lei et al. (2026) combined human metagenomic associations with mouse interventions using H₂S-producing *Desulfovibrio desulfuricans*, *phsABC*-engineered *Escherichia coli*, and sodium hydrosulfide (NaHS), linking microbial H₂S to hepatic peroxisome proliferator-activated receptor α (PPARα) suppression and metabolic dysfunction. These studies do not establish a coherent T2D-specific expression pattern across *dsrAB*, *aprBA*, *asrABC*, and organic-sulfur genes, nor a myocardial sulfur endpoint.

## Intestinal–hepatic first-pass processing constrains long-range sulfur signaling

Any gut-to-heart model must accommodate the chemical filtering between the colon and systemic blood. Quantitative estimates depend on specimen and assay. A human fecal study reported mean total acid-volatile sulfide of 0.66 μmol/g wet feces and measured H₂S of 0.17 μmol/g (Florin, 1991); fecal binding further limits the biologically available fraction (Jørgensen and Mortensen, 2001). By comparison, one mouse study measured free sulfide below 0.050 μmol/kg in blood and several tissues, including heart (Levitt et al., 2011). These matrix- and method-specific measurements illustrate compartmental differences, not a universal human concentration gradient or interchangeable measures of free and total sulfide.

Colonocytes provide a major first-pass sink. Sulfide:quinone oxidoreductase (SQOR, also termed SQR) initiates mitochondrial sulfide oxidation; persulfide dioxygenase (ETHE1), thiosulfate sulfurtransferase (TST/rhodanese), and sulfite oxidase (SUOX) support downstream sulfur processing (Landry et al., 2021). After intracecal H₂^35^S administration in rats, almost all absorbed label in cecal venous plasma was thiosulfate; systemic blood contained thiosulfate and sulfate, consistent with further hepatic or extrahepatic processing (Levitt et al., 1999). This supports first-pass conversion but not myocardial uptake of a particular carrier. In mice, intestinal epithelial SQOR deficiency combined with higher methionine intake increased serum thiosulfate, reduced goblet-cell density and mucus coverage, and produced systemic metabolic and brain phenotypes (Kumar et al., 2025). Thus, impaired clearance can couple sulfur exposure to barrier injury and potential inflammatory relays; the resulting cardiac effects remain untested.

Circulating sulfide is analytically labile. Acidification can release acid-labile sulfur and inflate estimates of free H₂S, while volatilization and oxidation can lower recovery; derivatization conditions and fluorescent-probe selectivity also affect results (Olson, 2012; Filipovic et al., 2018). Collection-to-quench time, pH, oxygen exposure, matrix recovery, and detection limits should therefore accompany quantitative values. Low circulating free sulfide and first-pass oxidation argue against assuming substantial intact gut H₂S delivery to cardiac cysteines. Alternatives include chemically defined oxidized or protein-bound sulfur carriers, sulfur-triggered endocrine or inflammatory signals, and altered endogenous cardiac sulfur synthesis. These possibilities imply that the biologically relevant mediator may not be free H₂S itself, but rather a reactive sulfur pool, sulfur-containing metabolite, or non-sulfur inter-organ signal that modifies cardiac sulfur homeostasis indirectly. Thiosulfate transport, myocardial uptake and conversion would each require testing; elevated serum thiosulfate alone is an exposure marker, not evidence of a causal cardiac carrier. No candidate relay is established as the dominant mediator. Germ-free comparisons show changes in circulating sulfur pools and host CSE activity, supporting microbiota-dependent regulation of host sulfur metabolism without distinguishing direct delivery from host adaptation (Shen et al., 2013).

## Microbiota-associated cardiac phenotypes: relevance and limits for sulfur signaling

Several DCM studies establish that gut microbial perturbation can influence cardiac phenotypes without demonstrating sulfur-specific mediation. Shi et al. (2025) integrated metagenomics, serum metabolomics, and cardiac transcriptomics in db/db mice, associating circulating carboxylic acids with cardiac PPAR-related transcriptional networks. In a type 1 diabetes model, Zheng et al. (2024) linked impaired microbial branched-chain amino acid (BCAA) degradation to hepatic PPARα–fibroblast growth factor 21 (FGF21) suppression and cardiac L-type amino acid transporter 1 (LAT1)/mechanistic target of rapamycin (mTOR) signaling; fecal microbiota transplantation and FGF21 knockdown strengthened causal inference. Myricetin and paeoniflorin studies connected microbiota remodeling to DCM phenotypes, with fecal transfer additionally supporting the myricetin study (Zhu et al., 2024; Wu et al., 2024). Their different diabetes models and non-sulfur mediators limit extrapolation to a common sulfur-specific DCM pathway.

Microbial sulfide can alter endocrine metabolism more directly. Qi et al. (2024) showed in male mice that microbiota-derived H₂S impaired intestinal L-cell mitochondrial respiration and lowered glucagon-like peptide-1 (GLP-1), worsening host metabolism. The study by Lei et al. (2026) supports a microbial H₂S–liver route through PPARα suppression. Together with the intestinal SQOR study (Kumar et al., 2025), these findings show effects beyond the lumen, but none measures source-resolved microbial sulfur delivery to myocardium or DCM persulfidation. Conversely, systemic NaHS improved metabolic measures alongside bacterial and fungal community changes in db/db mice (Liu et al., 2022). That donor-to-microbiota association neither establishes microbial mediation nor contradicts adverse effects of luminal microbial sulfide: source, exposure, and compartment differ. Collectively, these findings demonstrate that microbial sulfur metabolism can influence host physiology beyond the intestinal lumen, but they do not establish direct microbial sulfur delivery to myocardial cysteine residues. The heart should therefore be viewed as a responsive sulfur-signaling compartment whose reactive sulfur state may be regulated by both endogenous synthesis and systemic inter-organ signals.

## Cardiac reactive-sulfur signaling and persulfidation: a local endpoint, not source proof

In the diabetic heart, several studies report reduced CSE/H₂S-related signaling and beneficial effects of exogenous sulfide donors or compounds that modulate endogenous H₂S signaling on metabolism, proteostasis, cell death, and contractile function (Wu et al., 2017; Sun et al., 2018; Sun et al., 2019; Li et al., 2021; Gong et al., 2022; Liu et al., 2024). These experiments establish the cardiac sulfur system as pharmacologically modifiable, but they do not identify the physiological sulfur species responsible for the effect and do not demonstrate a microbial origin.

Three reported cardiac persulfidation candidates have been investigated across four cited DCM studies: muscle RING finger-1 (MuRF1) Cys44, synoviolin 1 (SYVN1/Hrd1) Cys115, and SUMO-specific protease 1 (SENP1) Cys683. Sulfide donor exposure has been associated with increased MuRF1 Cys44 persulfidation signals (Sun et al., 2020; Yu et al., 2020; Zhang et al., 2023; Peng et al., 2023). Hrd1 and SYVN1 denote the same E3 ubiquitin ligase. These reports associate sulfide donor exposure and cysteine mutation with altered proteostasis, lipid handling, or calcium homeostasis, but they represent an overlapping research program rather than independent confirmation from multiple laboratories. Most studies rely on modified biotin-switch approaches, which have important limitations in distinguishing persulfides from other reactive cysteine modifications. Sun et al. (2020) administered NaHS at 39 μmol/kg intraperitoneally every 2 days for 20 weeks; such intermittent donor exposure does not reproduce a measured physiological microbial sulfur flux. Although Zhang et al. (2023) also used GYY4137, sulfide donor solutions can generate reactive sulfur species during preparation and handling, complicating attribution to free H₂S itself (Filipovic et al., 2018). Therefore, these findings should be considered reported candidate-site observations, and independent replication using orthogonal site-resolved chemical approaches remains necessary.

The original methyl methanethiosulfonate (MMTS)-based modified biotin-switch has a problematic selectivity premise: persulfides can react with the blocking reagent, and signals may not distinguish competing cysteine redox states (Pan and Carroll, 2013; Zhang et al., 2014). Tag-switch approaches and low-pH quantitative thiol reactivity profiling provide complementary strategies, but sample handling, labeling efficiency, and site coverage still require controls (Zivanovic et al., 2019; Fu et al., 2020). Cysteine mutation does not establish chemical identity or occupancy and can alter basal protein function. Independent validation should combine orthogonal labeling, site-resolved liquid chromatography–tandem mass spectrometry (LC–MS/MS), controls for competing modifications, and functional rescue.

## Discussion and experimental roadmap

The evidence is best organized into three layers (Figure 1; Table 1). Established local or general processes include microbial sulfur production, intestinal sulfide oxidation, sulfur-responsive endocrine/metabolic effects, and cardiac sensitivity to manipulation of endogenous or donor-derived sulfur signaling. Cross-organ supporting evidence shows that microbiota-dependent metabolites can influence the diabetic heart through liver or systemic relays. The unresolved layer is the DCM-specific connection: the reviewed studies do not link a defined microbial sulfur gene and substrate to portal chemistry, a chemically identified systemic mediator, myocardial sulfur pools, site-specific persulfidation, and cardiac function in one source-resolved design.

**Table 1:** Representative evidence linking gut microbiota, microbial sulfur metabolism, reactive-sulfur signaling, and diabetic cardiomyopathy.

Evidence domain	Component or pathway	Main finding	Experimental context and limitation	Ref.
T2D context	Community functions	T2D microbiome associations; sulfate-reduction functions in Qin.	Human metagenomes; gene abundance is not expression or sulfur flux.	Qin et al. (2012); Mei et al. (2024)
Microbial genes	Inorganic/organic sulfur	Broad distribution of putative sulfidogenic genes.	Human gut datasets; capacity does not prove DCM-specific activity.	Braccia et al. (2021); Wolf et al. (2022)
Diet and ecology	Taurine respiration; assimilation	Bile acids favor *Bilophila*; *cysJ*/*cysM* manipulation changes sulfidogenesis.	Colitis-related studies; not cardiac evidence.	Devkota et al. (2012); Luo et al. (2024)
Host–microbe interaction	Systemic sulfur; donor effects	Microbiota status alters sulfur pools; NaHS alters bacterial/fungal communities.	Germ-free comparisons or db/db donor exposure; direction and mediation differ.	Shen et al. (2013); Liu et al. (2022)
Endocrine relay	Microbial sulfide-associated GLP-1 signaling	L-cell respiration and GLP-1 decrease.	Male mice; no myocardial sulfur or DCM endpoint.	Qi et al. (2024)
Hepatic relay	Microbial sulfide-associated PPARα signaling	*Desulfovibrio*, engineered *E. coli* and NaHS worsen metabolism.	Mouse interventions; no cardiac mediator identified.	Lei et al. (2026)
First-pass handling	Sulfide oxidation; SQOR	Cecal label appears as thiosulfate; intestinal SQOR loss alters distant physiology.	Rat tracing; diet-dependent mouse knockout; no causal cardiac carrier shown.	Levitt et al. (1999); Kumar et al. (2025)
DCM cross-organ support	Carboxylic acids; BCAA; microbiota	Cardiac associations and transfer/intervention effects.	db/db, T1D or diet/STZ models; non-sulfur evidence.	Shi et al. (2025); Zheng et al. (2024); Zhu et al. (2024)
Reported cardiac reactive-sulfur candidate	MuRF1 Cys44	Donor-responsive site linked to structural protein preservation.	Female db/db; NaHS; MMTS-based switch; orthogonal replication needed.	Sun et al. (2020)
Reported cardiac reactive-sulfur candidate	Hrd1/SYVN1 Cys115	Site linked to VAMP3–CD36 trafficking.	Both sexes included; NaHS; MMTS-based switch; sex-stratified validation limited.	Yu et al. (2020)
Reported cardiac reactive-sulfur candidate	SYVN1/Hrd1 Cys115	Site linked to SREBP1 degradation and lipid handling.	Female db/db; NaHS and GYY4137; MMTS-based switch.	Zhang et al. (2023)
Reported cardiac reactive-sulfur candidate	SENP1 Cys683	Site linked to SERCA2a SUMOylation and Ca^2+^ handling.	Donor-based db/db/cell study; chemical identity and occupancy need independent validation.	Peng et al. (2023)

The four cardiac reports have overlapping authorship and do not establish microbial sulfur provenance. Candidate-site findings represent reported associations rather than independent orthogonal chemical confirmation. Replication using site-resolved mass spectrometry and complementary chemical approaches is still required. BCAA, branched-chain amino acid; CD36, cluster of differentiation 36; DCM, diabetic cardiomyopathy; GLP-1, glucagon-like peptide-1; H₂S, hydrogen sulfide; MMTS, methyl methanethiosulfonate; MuRF1, muscle RING finger-1; PPARα, peroxisome proliferator-activated receptor α; SENP1, SUMO-specific protease 1; SERCA2a, sarcoplasmic/endoplasmic reticulum Ca^2+^-ATPase 2a; SQOR, sulfide:quinone oxidoreductase; SREBP1, sterol regulatory element-binding protein 1; STZ, streptozotocin; SUMO, small ubiquitin-like modifier; SYVN1/Hrd1, synoviolin 1; T1D/T2D, type 1/type 2 diabetes; VAMP3, vesicle-associated membrane protein 3.

Causality could be tested by deleting and rescuing defined microbial sulfur pathways while matching colonization burden. Stable-isotope tracing should follow sulfur through fecal, mucosal, portal, hepatic, systemic, and myocardial compartments. Oral ^34^S-labeled cysteine, methionine, or taurine can also enter host metabolism; sulfate can enter host sulfoconjugates. Bulk label incorporation therefore does not identify microbial sulfide. Microbial knockout/rescue controls and compartmental time courses are required. For myocardial persulfidation, the target should be the added sulfane-sulfur atom at a defined site rather than total ^34^S in newly synthesized protein.

Microbial manipulation should be crossed with cardiomyocyte-specific CSE loss- and gain-of-function to test dependence on endogenous synthesis. Residual CBS/3-MST activity and sulfur clearance must also be measured, because CSE deletion alone cannot establish sulfur provenance. Assays should separately resolve free sulfide, acid-labile sulfur, low-molecular-weight persulfides, thiosulfate, polysulfides, and protein-bound pools. Both sexes and prespecified treatment-by-sex analyses are needed: Qi et al. (2024) used male mice, whereas Sun et al. (2020), the anchor study reporting MuRF1 Cys44 persulfidation, used female db/db mice; Yu et al. (2020) included both sexes but did not establish a sex-resolved microbial sulfur–cardiac persulfidation pathway. Thus, both ends of the proposed axis currently rely on limited and heterogeneous sex designs, and no study has validated the complete pathway across sexes. The gap is heterogeneous sex inclusion and absent sex-stratified validation across the complete microbial sulfur–systemic mediator–cardiac endpoint sequence.

For translation, stool metagenomics/metatranscriptomics should be paired with dietary sulfur intake, bile-acid profiles, chemically resolved peripheral sulfur metabolomics, and cardiac phenotyping. Trimethylamine N-oxide (TMAO) remains a useful comparator because microbiota-dependent production of a defined circulating metabolite has been connected to cardiovascular risk in humans (Tang et al., 2013), but TMAO is not a sulfur-pathway intermediate in this model. Human organoid or microphysiological systems linking intestinal epithelium, liver, and cardiac tissue could provide an intermediate test of first-pass survival and target-organ effects before clinical claims are made.

Current evidence therefore supports a compartmental sulfur-relay hypothesis rather than an established linear gut-H₂S–heart axis. Progress will require source-resolved microbial genetics, quantitative first-pass sulfur chemistry, chemical identification of any circulating mediator, separation of microbial from endogenous cardiac sulfur, and orthogonal site-resolved verification of myocardial persulfidation.
